# ADVERSE CHILDHOOD EXPERIENCES PREDICTING INMATE STATUS AMONG OLDER COMMUNITY DWELLERS

**DOI:** 10.1093/geroni/igz038.1886

**Published:** 2019-11-08

**Authors:** Mai S Yang, Donald Hedeker

**Affiliations:** University of Chicago, Chicago, Illinois, United States

## Abstract

The goal of this study was to see if whether having exposure to early child adversity predicted inmate status among older adults. Very little research has focused on the relationship between inmate status and older adults, and factors that could predict someone being imprisoned. This study used data from the Health and Retirement Study (2006 – 2014) (N = 1,070) to examine this association. We used logit models to test if specific adverse childhood experience and race groups predicted inmate status among community dwellers age 55 and older (M = 72.2, SD = 8.5). More than half of the respondents were female (59%). About 1.9% respondent identified as non-Hispanic other, 7.2 % as Hispanics, 14.2% as non-Hispanic black, and 76.7% as non-Hispanic Whites. In terms of former inmate status 14% of the respondent indicated having been an inmate in jail or other correctional facility. Preliminary findings suggest predictors of inmate status were those who had trouble with the law in early adolescent years and those who self-reported as black. Findings from this study could provide insight into how early childhood experiences could predict inmate status in adulthood among older adults.

